# Analysis of vocal communication in the genus *Falco*

**DOI:** 10.1038/s41598-023-27716-y

**Published:** 2023-02-01

**Authors:** Carole S. Griffiths, Neil L. Aaronson

**Affiliations:** 1 LIU Brooklyn, Brooklyn, NY 11201 USA; 2 Department of Ornithology, American Museum of Natural History, New York, NY 10024 USA; 3 Physics Program, Stockton University, Galloway, NJ 08205 USA

**Keywords:** Acoustics, Animal physiology

## Abstract

Vocal learning occurs in three clades of birds: hummingbirds, parrots, and songbirds. Examining vocal communication within the Falconiformes (sister taxon to the parrot/songbird clade) may offer information in understanding the evolution of vocal learning. Falcons are considered non-vocal learners and variation in vocalization may only be the result of variation in anatomical structure, with size as the major factor. We measured syringes in seven *Falco* species in the collection at the American Museum of Natural History and compiled data on weight, wing length, and tail length. Audio recordings were downloaded from several libraries and the peak frequency and frequency slope per harmonic number, number of notes in each syllable, number of notes per second, duration of each note, and inter-note duration was measured. Mass, wing length, and syringeal measurements were strongly, positively correlated, and maximum frequency is strongly, negatively correlated with the size. Frequency slope also correlates with size, although not as strongly. Both note and inter-note length vary significantly among the seven species, and this variation is not correlated with size. Maximum frequency and frequency slope can be used to identify species, with the possibility that bird sounds could be used to identify species in the field in real time.

## Introduction

Vocalizations are the major method used for communication by birds, one of several ways that birds communicate (e.g. behavioral, morphological, and vocal). Bird vocalizations have been generally divided into two types: songs and calls. Calls are simpler and often innate, whereas songs are more complex and usually involve a period of learning the song and a period of practice to produce the song.

**Figure 1:**
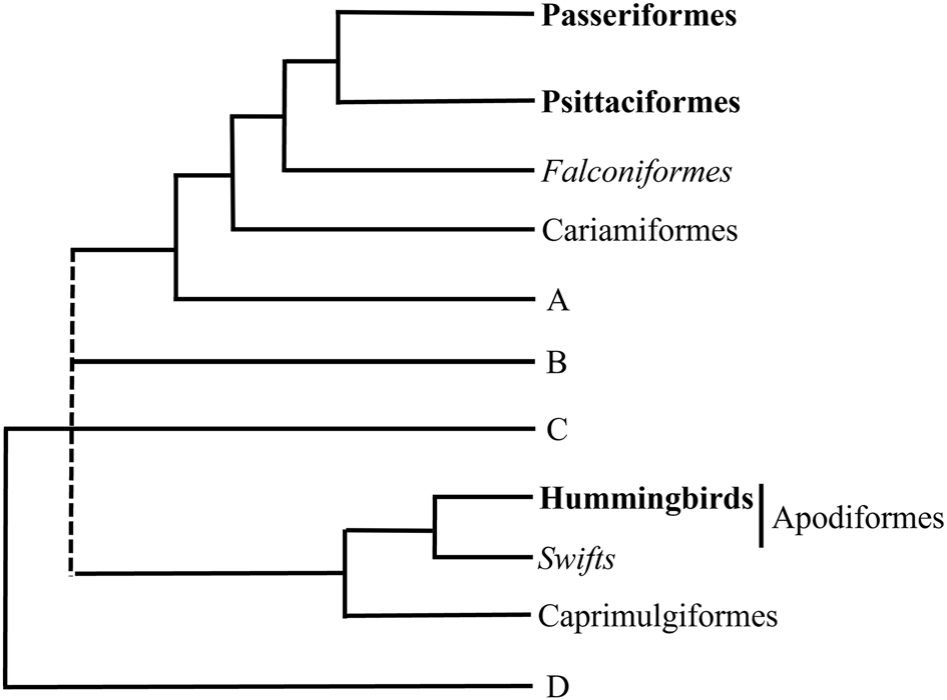
Avian phylogeny (adapted from Hackett et al.^7^ and Braun and Kimball^8^). Clades of vocal learners are in bold, sister taxa to learners are italicized, all other orders are in roman text. Clades B, C and the Apodiformes are in an unresolved relationship with the Passeriformes clade. D includes the Galliformes and Anseriformes, sister taxa to all of the others in this phylogeny. See Hackett et al. (2008) for clades included in A, B, C, D.

This ability to produce complex songs through vocal learning is believed to occur in only three groups of birds—the oscine Passeriformes, the Psittaciformes (sister taxon to the Passeriformes) and the Trochilidae (hummingbirds)^1,2^, although there is evidence that suboscine Passeriformes may learn songs^3–6^. This raises the question, how has vocal learning evolved in birds? To answer this question, it would be helpful to have an unambiguous definition of vocal learning, and to analyze vocal communication in close relatives of the three groups of learners (e.g., the swifts, sister taxon to hummingbirds, and the Falconiformes, sister taxon to the clade of parrot and songbirds, Fig. 1)^7,8^.

One well-studied definition of vocal learning in the three groups is the ability of birds to modify songs as a consequence of hearing those songs. This has been specifically termed vocal production learning (VPL)^9–11^. The ontogeny of VPL has been characterized as a multi-part process with a sensitive learning phase in which young birds hear appropriate songs, followed by time to establish a neural memory of the song, and ending with a practice period to produce and refine the learned song^12–14^. Non-learners can produce their species song without hearing it, and may not possess the necessary forebrain neural circuitry to learn^5^.

There are more general definitions: (1) usage learning (individuals learn a context in which to produce specific vocalizations), and (2) comprehension learning (individuals learn to modify a response to a heard vocalization)^9–11^. These have wider taxonomic occurrences. For example, male prairie chickens on their display grounds learned a new three-note call from a hybrid, and captive males housed with the hybrid used that vocalization, and a male sharp-tailed grouse learned an altered coo that had been played eight times^15^.

In addition, each of these types of learning could be accomplished without the considerable neural and anatomical adaptations that underlie vocal production learning. The neural adaptations include acquisition of specific brain nuclei related to the two pathways—anterior nuclei in the learning pathway and posterior nuclei in the production pathway (with variations among the three vocal learners)^16^.

**Figure 2:**
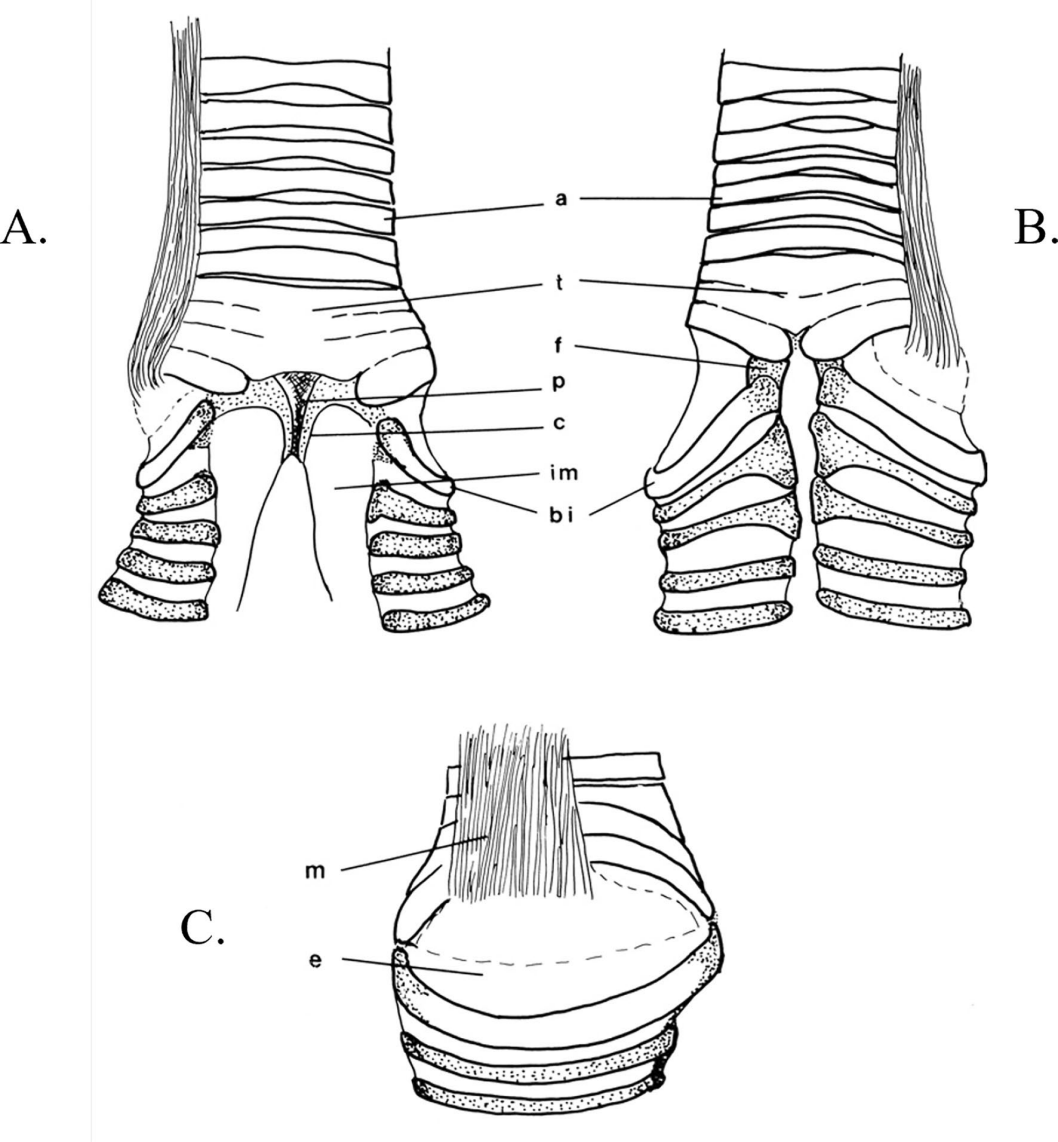
*Falco berigora* syrinx (adapted from Griffiths^18^). (**A**) Ventral view. (**B**) Dorsal view. (**C**) Lateral view. Abbreviations: **a** (ossified tracheal elements), **bi** (first bronchial ossified half-ring, the more caudal bronchial half-rings are cartilaginous), **c** (cartilaginous border), **e** (lateral tympaniform membrane), **f** (fused ends of the first tracheal element (A1) and first bronchial half-ring (B1), **im** (medial tympaniform membrane), **m** (*M. tracheolateralis*, illustration on one side only), **p** (pessulus). Stippling indicates cartilaginous tissue, cross-hatching indicates dense ossified tissue.

Anatomical adaptations involve the avian syrinx, the sound producing organ, illustrated for the relatively simple syrinx of *Falco berigora* (Fig. 2). Ames’^17^ definitions of syringeal components are used. These consist of rings on the trachea and bronchi; the tympanum, fused and ossified tracheal rings at the trachea-bronchial junction; the pessulus, a cartilaginous or ossified bar in the mid-sagittal plane of the trachea between the bronchi; and two types of membranes, the medial tympaniform membranes on the medial surface of the bronchial tubes, extending caudally from the pessulus, and the lateral tympaniform membranes on the lateral walls of the bronchi. Finally, there are two muscles that originate outside the syrinx, the extrinsic muscles *Muscularis (M.) tracheolateralis* and *M. sternotrachealis*^18^.

Birds that are vocal production learners have additional muscles that are intrinsic to the syrinx, varying from two in the Psittaciformes^19^, three in the vocal-learning hummingbirds^20^, and up to six in the Passeriformes^21^. In addition, species within the oscine Passeriformes and vocal-learning hummingbirds have a thickened area in their lateral membranes (the labia) to which the intrinsic muscles attach. These are not present in the Psittaciformes^20^.

To determine if adaptations arose independently in the three vocal production learners, it may be helpful to consider evolution of vocal learning as a gradual, rather than as a binary process^22,23^. For example, there may be levels of learning in VPL birds^14^ and possible VPL in two species of bellbirds (suboscine Passeriformes), based on behavior and genetic analysis^4,6^. Finally, rudimentary neural structures related to those of vocal learners have been found in two other species of suboscines, the Eastern phoebe^5^ and the scale-backed antbird^3^.

The Falconiformes is sister taxon to the clade containing two of the three vocal learners, the Psittaciformes and Passeriformes (Fig 1)^7,8^. Falcons may not have the necessary neural connections (either complete or rudimentary) and they do not have the intrinsic syringeal muscles necessary for vocal learning (Fig. 2)^18,19,24^. Their vocalizations are relatively simple and are generally regarded as calls, not songs. However, because the definitions and distinction between calls and songs are not always clear (e.g. Borror 1975^25^), we are using “song” for falcon vocalizations and “learned song” for the vocal learners.

For falcons, variation in song structure may be relatively minor and song should not be a selective factor in their evolution. Distinctions have usually been based on behavioral context rather than on spectral and temporal features, and vocalizations have generally been defined phonetically. For example, three or four different songs have been proposed for several falcon species, each song used for specific behaviors, the klee or kak (e.g. aggressive behaviors), chitter (breeding behavior) , chip (food transfer, courtship displays) and whine (food begging, female solicitation^26–28^). The klee is probably the most familiar to anyone who has heard a peregrine, kestrel, or merlin. For the black falcon (*F. subniger*) and lanner falcon (*F. biarmicus*), around 10 song types had been described.

There have been a few detailed analyses of sound. In the American kestrel (*F. sparverius*), differences in vocalizations between male and female, and nestling and adult were analyzed by Smallwood and Dudajek^29^. Their study indicated that nestlings’ calls became more complex in harmonic structure, and increased in number of notes per call and rate. Nestlings developed the adult klee in about two weeks. However, their analysis could not distinguish calls based on sex. In the peregrine falcon (*F. peregrinus*), relative frequency and context of vocalizations varies with sex and subspecies. Male calls were higher in frequency, and vocalizations of the two sexes could be distinguished fairly accurately. However, no geographic differences were found among the four subspecies observed in captivity. In general, these are broad characterizations and there have been no detailed, comparative examinations of the elements of falconid song.

We were interested in investigating the evolution and development of vocal communication within the Falconiformes, and whether this will provide insight into how vocal learning has evolved in birds. As an initial study, we examined the structure of vocalizations in seven falcon species: the merlin (*F. columbarius*), American kestrel, peregrine falcon, nankeen kestrel (*F. cenchroides*), aplomado falcon (*F. femoralis*), brown falcon (*F. berigora*), and lanner falcon (*F. biarmicus*). If these species are not vocal learners, variation in vocalization may be mainly related to variation in anatomical structure, in particular the syrinx. For these seven species, syringeal structure is relatively invariant, size being the only variation . Our null hypothesis, therefore, is that size would be the major factor in variation in vocal communication in these species and that there would be a correlation between sound and syringeal structure.

## Methods

### Acoustic measurement

Recordings for the seven species were obtained from three major sources: the Macauley Library in the Cornell Lab of Ornithology, the Borror Laboratory of Bioacoustics at Ohio State University, and Xeno-Canto (xenocanto.org). Whenever possible, audio files in WAV format were obtained. In the analyses, we focused on two different areas: variation in frequency, and variation in the structure of the song.

#### Acoustic spectral analysis

Based on detailed analysis, 29 audio recordings of the species studied were processed and analyzed (the source, recording ID, sex, and age range for each audio sample can be found in Table 1). Each recording was high-pass filtered with a − 3 dB cutoff at 100 Hz. Each was then noise-reduced using a Weiner filter, where sections of the recordings containing only background noise were used to estimate the noise power^30^. Recordings for which clearly readable harmonic structure in the sonogram could not be produced (due to, for example, a low signal-to-noise ratio) were rejected.

**Table 1:** Recordings used for frequency and song structure analysis.

Species	Museum	ID	Sex	Age
*F. berigora*	Macauley	151339$^1$	Female	Adult
		154127	Female/male	Adult
		156465$^*$	Unknown	Juvenile
		157722	Unknown	Juvenile
*F. biarmicus*	Macauley	4392	Female	Adult
		89506$^1$	Unknown	Adult
		94053$^*$	Female	Adult
*F. cenchroides*	Macauley	149826$^1$	Female	Adult
		150163	Unknown	Unknown
		150266	Male	Adult
		154151$^1$	Male	Adult
*F. columbarius*	Macauley	4408	Unknown	Unknown
		4409	Female	Juvenile
		105837	Unknown	Adult
	Xeno	XC36360	Unknown	Unknown
		XC10564$^*$	Male	Adult
		XC137949	Female	Adult
		XC137975	Female	Adult
		XC14514$^*$	Unknown	Adult
		XC175204	Male	Juvenile
	Borror	BLB2127$^*$	Female	Adult
		BLB23867	Male	Adult
		BLB2768$^*$	Female	Adult
		BLB3917$^*$	Male	Adult
*F. femoralis*	Macauley	163292$^*$	Female/male	Adult
		163293$^*$	Female/male	Adult
		163294$^*$	Female/male	Adult
		195472	Female	Adult
	Xeno	XC484661	Male	Adult
		XC50280	Unknown	Unknown
		XC53276	Female	Adult
		XC53277	Female	Adult
		XC65472$^*$	Unknown	Adult
		XC17439$^*$	Unknown	Adult
*F. peregrinus*	Macauley	44151	Female	Adult
		229201	Unknown	Juvenile
		1375731	Male and female	Adult
	Xeno	XC35917$^*$	Female	Adult
		XC35979$^*$	Female	Adult
		XC13332$^*$	Unknown	Adult
	Borror	BLB16715	Unknown	Adult
		BLB1671$^*$	Unknown	Adult
		BLB2226$^*$	Unknown	Nestling
		BLB2226$^*$	Female	Adult
*F. sparverius*	Macauley	4423	Female and male	Adult
		59860$^*$	Male	Adult
		59861$^*$	Male	Adult
		1202081	Unknown	Unknown
		107056$^*$	Female	Adult
		107989$^*$	Female	Adult
		133146$^*$	Unknown	Unknown
		171944$^*$	Female	Adult
	Borror	BLB6051	Unknown	Adult
		BLB1033$^*$	Female and male	Adult
		BLB2225$^*$	Unknown	Juvenile
		BLB2988$^*$	Unknown	Adult
		BLB2988$^*$	Male	Adult

$^*$Used only for song structure analysis.

$^1$Used only for frequency analysis.

**Figure 3:**
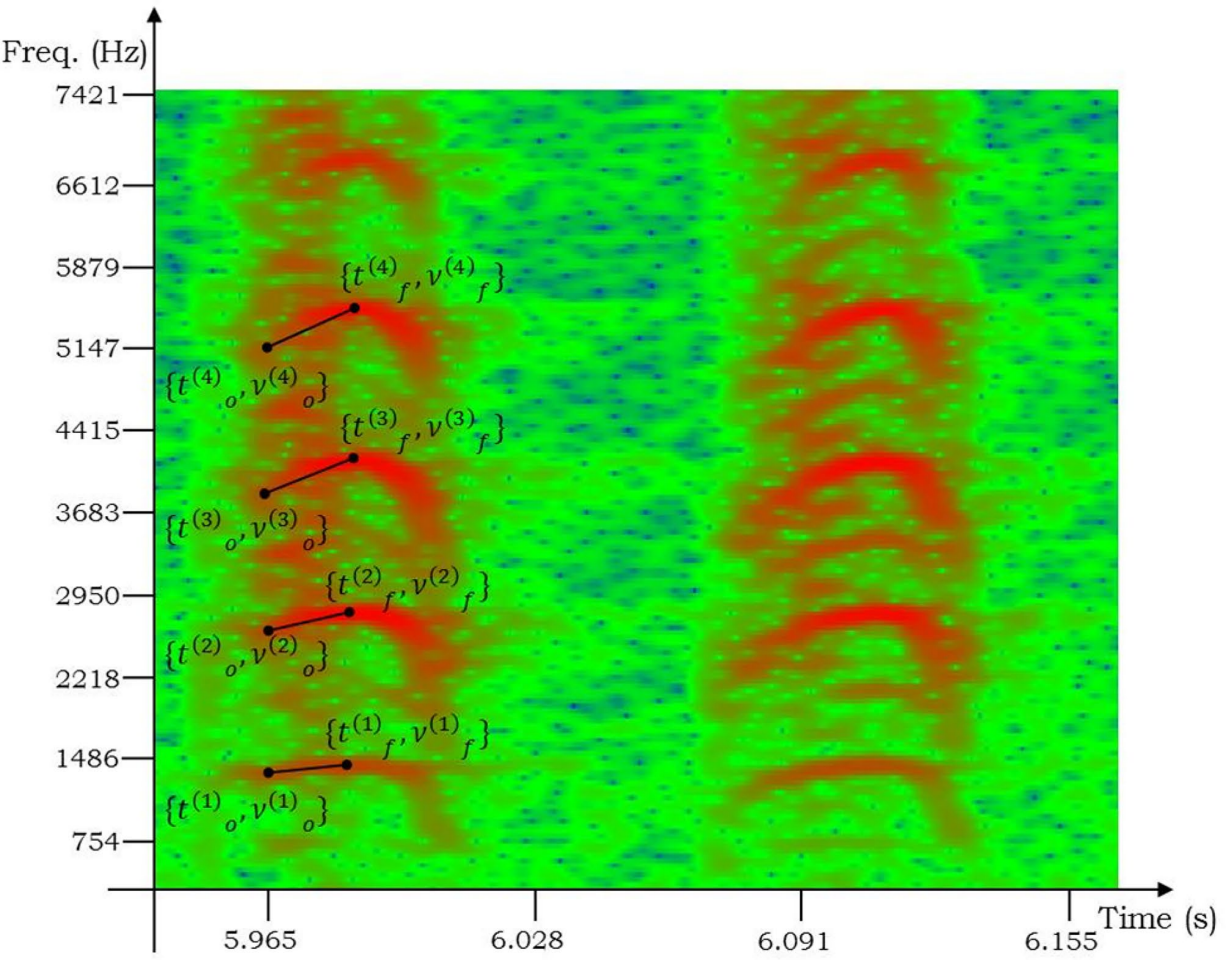
Two klee notes from a recording of the *F. femoralis* (aplomado falcon). Several harmonics are visible (red) for each. The initial time $t_o^{(n)}$, time at peak frequency $t_f^{(n)}$, initial frequency $\nu _o^{(n)}$, and peak frequency $\nu _f^{(n)}$ within each harmonic *n* are labeled for the first klee.

For analysis, a sonogram was generated using a 1024-point FFT (Blackman-Harris windowed, 99% overlap). Figure 3 shows a sonogram of two consecutive notes within a klee for one specimen of the aplomado falcon. For each note, several harmonics are visible, appearing in red. The frequency modulation is approximately linear with a positive slope, followed by a shorter linear modulation with a negative slope. We measured the positive-sloped linear frequency modulation. The harmonic numbers are denoted by *n*. The onset time and frequency ($t_o^{(n)}$ and $\nu _o^{(n)}$, respectively), as well as the time at which the peak frequency is reached and the peak frequency itself ($t_f^{(n)}$ and $\nu _f^{(n)}$, respectively) were measured manually and recorded for each harmonic. These points are depicted for the first four harmonics of one note in Fig. 3. Harmonics up to the sixth were measured, when such frequency data were available and clear in the recordings. Since each harmonic frequency component should be part of a harmonic series, each harmonic can provide an estimate of the peak frequency and frequency slope of a note, thus leading to a more accurate estimate of these measures within each note.

For each usable recording, at least 12 and as many as 24 notes were analyzed. For each note, time and frequency data for as many harmonics as possible were recorded. In some recordings, fundamental frequency ($n=1$) data were not readable on the sonogram either because of the original recording method or background noise made it impossible to do so. In many recordings, higher harmonics (typically $n\ge 4$) were also unavailable, often due to the fidelity of the original recording. In general, the spectral features of higher harmonics are more pronounced, which often made finding the points of maximum frequency easier. However, the energy in higher harmonics also tended to fall off quickly and become indistinguishable from the background, making starting times and frequencies difficult to distinguish. Fortunately, estimation of the slope of a line does not rely on choosing any particular start and end points.

For each harmonic of each note, two quantities were of interest: the peak frequency per harmonic number $(\nu _{f}^{(n)}/n)$ and the frequency slope per harmonic number. The frequency slope for the $n{\text {th}}$ harmonic $(f\!s_n)$ is the rate of linear frequency modulation, calculated simply here as:

$$\begin{aligned} f\!s_{n}=\left( \frac{\Delta \nu }{\Delta t}\right) _{n}=\frac{ \nu _{f}^{(n)}-\nu _{o}^{(n)} }{ t_{f}^{(n)}-t_{o}^{(n)} } \end{aligned}$$

Since each harmonic should be a multiple of the fundamental frequency, both the peak frequency per harmonic number and frequency slope per harmonic number $(f\!s_{n}/n)$ should be roughly equal within each note, but may very across notes.

#### Acoustic temporal analysis

For this analysis, only those sonograms that could be characterized as the klee or kak song were used (Table 1). Sonograms were processed with Raven Pro 1.5 (www.birds.cornell.edu/raven) and four variables were measured: number of notes in each song, number of notes per second, duration of each note, and inter-note duration.

Analyses of variation within and among individuals within a species, and among all species were calculated for both note and inter-note duration. For the analyses within and among individuals in a species, we used the data for each note and each inter-note. For the analysis among species, we used the averages for each individual in a species. Regression analyses were performed for the note and inter-note duration vs. wing length, weight and the seven syringeal structures.

### Anatomical measurements

All anatomical measurement were made on specimens in museum collections—no live birds were used for this research. We measured syringes in the 18 specimens available in the syringeal collection at the American Museum of Natural History (Table 2). Our choice of taxa was limited by the availability of syringeal specimens and vocalizations. Our measurements focused on the two major structures of the falconid syrinx, the tympanum and the lateral membrane. Measurements included the TWJ—posterior tympanum width at the base of the tympanum, TWT—anterior tympanum width from the most rostral fused tracheal element, TLJ—posterior tympanum depth dorsal to ventral from the end of the most caudal fused tracheal element, TLT—anterior tympanum depth, LTM—the width of the lateral tympaniform membrane dorsal to ventral from the ends of the membrane, THD and THV—dorsal and ventral height of the tympanum at the midpoints of the fused elements (Fig. 2).

**Table 2:** Syringeal measurements (mm) of *F. peregrinus* (FP), *F. columbarius* (FC), *F. sparverius* (FS), *F. cenchroides* (FCen), *F. berigora* (FBe), *F. femoralis* (FF), and *F. biarmicus* (FBi).

	ID	Sex	TWJ	TWT	TLJ	TLT	THV	THD	LTM
FP	19751	F	7.50	5.5	6.5	5	4.50	4.5	7.5
FP	8499	F	7.80	5.8	6.5	5.5	6.00	5.5	8.5
FC	14713	M	4.40	4	4.1	3.7	3.00	2.8	4.75
FC	19752	F	5.00	4.25	4.5	3.75	2.50	3	5
FC	23157	F	5.60	5	4.8	4	4.00	4.75	5.5
FS	1217	F	4.00	3.3	3.15	3	2.00	2	4
FS	8688	F	4.00	2.75	3	2.75	2.00	2	3.75
FS	csg21	F	3.00	2.25	3	2.5	2.00	1.75	3.75
FS	pk429	F	3.75	2.75	3.25	2.5	1.50	2.5	3.5
FS	pfc432	F	3.25	2.5	3	2.5	1.00	1.5	3.5
FS	csg9210	M	3.50	3	2	1.75	1.00	1.25	3.5
FS		M	3.50	3	3	2	1.25	2	3.75
FS	8413	M	2.50	1.5	3	2.5	1.00	1.5	3.5
FS	8430	M	3.75	3	3	2	1.00	1.5	3.75
FCen	193394		4.51	3.46	3.22	3	3.92	3.9	4.35
FBe	193358		6.36	4.95	5.96	4.5	4.72	5.1	7.26
FF	LSU123309		5.66	4.55	5.4	4.4	3.1	4.63	5.5
FBi	15927	M	7.41	5.88	6.19	4.6	4.23	4.64	7.84

TWJ = posterior tympanum width, TLJ = posterior tympanum depth; TWT = anterior tympanum width, TLT= anterior tympanum depth; THD and THV = medial height of the tympanum dorsally and ventrally; LTM = width of lateral tympaniform membrane.

Because there were no skin specimens for the syringeal specimens, and because skin specimens are not used for determining weight, we used data compiled from Brown and Amadon^31^, Johnsgard^32^, and Clark and Wheeler^33^ for estimates of weight, wing length, and tail length for these species (Table 3). Pearson’s tests of linear correlation were performed for the three anatomical measurements (weight, tail and wing length). We ran the tests multiple times using the lowest, highest and average or medial measurement of weight, wing length and tail length for each species, and then used the average measurement for the final analysis. Finally, regression analyses for the seven species were performed for the correlation of syringeal measurements with weight and with wing length.

**Table 3:** Weight (g), wing and tail length (mm) of *F. peregrinus* (FP), *F. columbarius* (FC), *F. sparverius* (FS), *F. cenchroides* (FCen), *F. berigora* (FBe), *F. femoralis* (FF) and *F. biarmicus* (FBi).

	Weight^32^	Avg.	Weight^33^	Avg.	Wing Lgth.	Avg.	Tail Lgth.	Avg.
FP male	550–647	611	453–685	581	301–327	314	138–154	145
FP female	825–1094	952	719–952	817	340–376	356	167–192	179
FC male	129–187	155	129–187	155	182–200	189	114–128	121
FC female	182–236	210	182–236	210	192–215	208	120–140	134
FS male	94–126	114	94–126	114	174–198	183	116–147	129
FS female	132–160	147	132–160	147	178–207	195	119–142	130
FCen male	121–195				237–255		146–168^31^	
FCen female	115–273				255–275		151–176^31^	
FBe male	387–512		316–390^31^		319–355		169–230^31^	
FBe female	505–635		430–860^31^		350–397		171–233^31^	
FF male		235	208–305	265	248–267	257	172–193	182
FF female	271–305		310–460	391	272–302	290	192–207	199
FBi male	450–650	550			308–332	317	160–178	
FBi female	550–800	650			340–360	350	185–210	

## Results

### Acoustic spectral analysis

For two species (brown falcon and lanner falcon), available recordings did not have high enough signal-to-noise ratios to obtain frequency data. Therefore, frequency analyses were performed for five of the seven species. There are clear differences among the five species, both in the peak frequency per harmonic of their notes (Table 4), and in the frequency slope (Table 5; one-way ANOVA, $d\!f=629$, $F=1734$, $p<0.001$). In short, the larger the bird, the lower the fundamental frequency and the slower the rate of change in frequency (smaller frequency slope) within notes.

**Table 4:** Maximum frequencies per harmonic number $\nu _{f}^{(n)}/n$ (in Hz) for each species.

	$\nu _{f}^{(1)}$	$\nu _{f}^{(2)}/2$	$\nu _{f}^{(3)}/3$	$\nu _{f}^{(4)}/4$	$\nu _{f}^{(5)}/5$	$\nu _{f}^{(6)}/6$	Average
*F. sparverius*	2460 ± 70	2460 ± 67	2440 ± 60	2430 ± 66	2460 ± 75		2450 ± 30
*F. columbarius*	2230 ± 60	2210 ± 62	2190 ± 54				2210 ± 34
*F. peregrinus*	1230 ± 61	1250 ± 75	1250 ± 76	1240 ± 81			1240 ± 37
*F. femoralis*	1410 ± 40	1410 ± 45	1400 ± 44	1400 ± 45	1400 ± 38	1390 ± 34	1400 ± 17
*F. cenchroides*	2210 ± 100	2180 ± 120	2170 ± 110	2160 ± 120			2180 ± 57

Errors are 95% confidence intervals.

**Table 5:** The frequency slope per harmonic number $f\!s_{n}/n$ (in kHz/s) for each species.

	$f\!s_1$	$f\!s_{2}/2$	$f\!s_{3}/3$	$f\!s_{4}/4$	$f\!s_{5}/5$	$f\!s_{6}/6$	Average
*F. sparverius*	26 ± 8.2	26 ± 5.8	26 ± 4.6	24 ± 3.9	25 ± 3.0		25 ± 2.4
*F. columbarius*	11 ± 2.5	9.5 ± 2.9	9.1 ± 2.2				10. ± 1.5
*F. peregrinus*	4.0 ± 1.1	3.5 ± 0.82	3.4 ± 0.77	3.4 ± 1.0			3.6 ± 0.47
*F. femoralis*	3.3 ± 0.53	3.4 ± 0.62	3.4 ± 0.62	3.2 ± 0.47	3.1 ± 0.49	3.0 ± 0.48	3.2 ± 0.22
*F. cenchroides*	6.5 ± 1.2	6.5 ± 1.1	6.6 ± 1.1	6.2 ± 1.1			6.4 ± 0.56

Errors are 95% confidence intervals.

There is strong correlation between frequency slope ($f\!s$) and two of the syringeal structures (the height of the tympanum dorsally and ventrally, i.e. the number of fused tracheal rings) and weaker correlation between wing and tail length, and all of the other syringeal structures. Finally, there are stronger associations between peak frequency per harmonic number $(\nu _{f}^{(n)}/n)$ and wing length, and all but one of the syringeal structures (Table 6).

**Table 6:** Pearson’s product-moment correlation coefficient (*r*) between the physical measurements and the frequency parameters (average frequency slope per harmonic number $f\!s$, and frequency maximum per harmonic number $\nu _f$).

	Avg. $f\!s$	Avg. $\nu _{f}$
Avg. mass	− 0.553	− 0.773
Avg. wing length	− 0.760	− **0.902**
Avg. tail length	− 0.724	− 0.770
TWJ	− 0.751	− **0.910**
TWT	− 0.785	− **0.866**
TLI	− 0.703	− **0.933**
TLT	− 0.776	− **0.926**
THV	− **0.827**	− 0.697
THD	− **0.974**	− **0.881**
LTM	− 0.654	− **0.874**

Instances for which $r>0.800$ are highlighted in bold. For the syringeal measurements: TWJ = posterior tympanum width, TLJ = posterior tympanum depth; TWT = anterior tympanum width, TLT = anterior tympanum depth; THD and THV = medial height of the tympanum dorsally and ventrally; LTM = width of lateral tympaniform membrane.

### Acoustic temporal analysis

For these analyses, we first evaluated all seven species and then, because of limited data, we performed the analysis on five of the seven (eliminating the brown falcon and lanner falcon as above). There was a significant difference among the species in both the length of the notes in the song ($p<0.001$ for the seven and five species analysis), and the length of the internote ($p=0.00137$ and $p<0.001$ for the seven and five species analysis, respectively).

There is also a significant difference within six of the species for the length of the notes ($p<0.001$; lanner falcon, $p=0.668$). However, for this species there were only two sonograms analyzed, and within these sonograms, the number of songs was limited. There are significant differences of inter-note duration only within four of the seven species (brown falcon, $p=0.188$; nankeen kestrel, $p=0.526$; peregrine falcon, $p=0.167$).

Regression analyses of note length vs. LTM or wing resulted in $R^2$ values of 0.00726 and 0.1656. Analyses of inter-note length produced $R^2$ values of 0.369 and 0.404, higher than those of note length, but not indicative of significance. Reducing the analyses to five species led to higher values, but still not approaching statistical significance.

### Anatomical measurements

The basic structure of the syrinx and the syringeal muscles are the same for all of these species (Fig. 2). Differences only occur in the amount of ossification of some of the tracheal and bronchial rings (among and within species), and in size. Syringeal measurements of the seven species are illustrated in Table 2. Sex differences are reported for the merlin and American kestrel, the only species for which there are these data. There is overlap in individual males and females within the American kestrel, however the average measurements for males are smaller than those of females for the six tympanum structures but not for the lateral membrane. Six of the seven measurements of the two female merlins are larger than those of the male. For this species, the height of the male tympanum ventrally was intermediate to the two females.

Available measurements for mass, wing, and tail length for the seven species are detailed in Table 3. There is a general trend from large to small, with the peregrine falcon, lanner falcon and brown falcon the three largest, and the American kestrel, the smallest. But there is some overlap in wing length and mass. The peregrine falcon has the greatest mass, but the brown falcon the longest wing length. In all, males are smaller than females, as is expected with raptors.

Regression analyses on the measurements indicated a strong correlation between mass and wing length $(r=0.96)$, and a weaker correlation between mass and tail length $(r=0.71)$, and between wing and tail length $(r=0.79)$. There is also a strong correlation between each of the seven syringeal structures and both mass and wing length, with the wing length correlation slightly stronger except for the tympanum ventral height measurement (Table 7).

**Table 7:** Pearson’s product-moment correlation coefficient (*r*) between the average wing length and mass, and measurements of seven syringeal structures.

	Wing length	Mass
TWJ	0.94	0.92
TWT	0.844	0.814
TLI	0.87	0.852
TLT	0.919	0.882
THV	0.855	0.891
THD	0.88	0.815
LTM	0.921	0.919

TWJ = posterior tympanum width, TLJ = posterior tympanum depth; TWT = anterior tympanum width, TLT= anterior tympanum depth; THD and THV = medial height of the tympanum dorsally and ventrally; LTM = width of lateral tympaniform membrane.

## Discussion

This was an initial analysis of song structure in seven falcon species to provide a baseline for investigating evolution and development of vocal communication within the Falconiformes (sister taxon to Psittaciformes and Passeriformes). We limited this initial baseline study to the analysis of seven *Falco* species similar in anatomy and in syringeal morphology, to determine if there were parameters of variation at this level that would form the basis of our future research.

Avian vocalizations are produced through vibrations of syringeal membranes as air flows through the syrinx. It is commonly understood that the complexity of learned song requires neural modifications and intrinsic syringeal muscles acting on the syringeal labia/membranes. Because the falcon syrinx does not have the intrinsic syringeal muscles considered necessary for complex, learned song^19,34,35^, our hypothesis was that variation in sound production in falcons would be correlated with size.

As might be expected, we found that those characteristics of falcon vocalization that vary with the body size of the bird are those that relate to the frequency of sound. Thus, maximum frequency is strongly, negatively correlated with size of the bird, both for LTM, wing, and mass—the larger the bird, the lower the peak frequency. In addition, frequency slope also correlates with the size of the bird, although not as strongly.

Contrary to frequency, the structural characteristics of song syllables are not associated with size. Both note and inter-note length vary significantly among the species and this variation is not correlated with size. Similarly, the length of the note within species varies significantly for six of the seven species, but not for the lanner falcon. However, for this species there were only two sonograms analyzed, and within these sonograms, the number of songs was limited. For most species, this note-length variation occurs within syllables, usually with a far longer initial note. The inter-note length varies significantly within only four of the seven species (lanner falcon, aplomado falcon, merlin, and American kestrel).

Vocal learning is currently thought to occur in only three clades. As noted above, the evolution of vocal learning may proceed in a gradual, step-like process^22,36^. For example, there are scattered occurrences of vocal learning in other orders of birds^36^. And, among the three clades of vocal learners there is variation in the neural and anatomical modifications necessary for learned song. Hummingbirds and oscine songbirds have medial membranes and labia in the lateral membranes that produce variation, and their membranes occur on the bronchi, giving them two pairs of possible sources of sound variation. They also have three to six pairs of intrinsic syringeal muscles. Parrots produce complex songs, yet have simpler syringes, with membranes on the tracheal portion of the syrinx (with one pair of source sources), and only one pair of intrinsic muscles.

Because the anatomical modifications in parrots are simpler than in songbirds, analysis of the Falconiformes, whose syringes lack intrinsic muscles, may provide clues into the evolution of song and of learned song. Our results indicate that, at a minimum, analyzing details of song structure within the Falconiformes may illuminate evolution of vocalization in this order. And this kind of spectral-temporal analysis may uncover more subtle variations that provide increased understanding of the evolution of vocal learning in birds (e.g. ten Cate^36^).

This was an initial baseline study to determine the kinds of variation in falcon vocalizations. We limited the analysis to *Falco* species because of the similarity of the species in anatomy and in syringeal morphology. Of the types of vocalizations characterized for these species, we examined the ‘klee’ vocalization only, the vocalization that is most common. However, when examined in detail, the notes in the ‘klee’ are different in each of these species, and in the juvenile vs. adult birds that we were able to study. In addition, the range of vocalizations among the species we analyzed vary beyond the klee, chitter, chip, and whine, including combining aspects from different songs (e.g. combining the klee and whine, or the whine and chitter). We intend to expand this research to the analysis of the range of species vocalizations, and to the analysis of species in all genera in the Falconiformes, looking in particular at the syllable and note structure for those species’ vocalizations.

One potentially useful result of this research is that the maximum frequency and frequency slope can be used to more accurately identify species. These parameters can perhaps be quantified using mobile phones, for which spectrum analysis apps already exist, or may improve the accuracy of existing methods. This has potential application as well for the automation of ecological studies such as wildlife assessment surveys for environmental assessment reports such as those required prior to the start of construction activities.

## Data Availability

All data generated or analysed in the course of this study are included in this article or are available from the corresponding author upon reasonable request.
